# SOD3 and eNOS genotypes are associated with SOD activity and NO_x_

**DOI:** 10.3892/etm.2014.1720

**Published:** 2014-05-19

**Authors:** XIAOLONG DONG, DEJUN LI, HONG LIU, YANYAN ZHAO

**Affiliations:** 1 Department of Clinical Genetics, Shengjing Hospital of China Medical University, Shenyang, Liaoning 110004, P.R. China; 2 Department of Medical Genetics, China Medical University, Shenyang, Liaoning 110001, P.R. China

**Keywords:** nitric oxide synthase, oxidative stress, polymorphism, superoxide dismutase, hypertension

## Abstract

Oxidative stress, characterized by increased reactive oxygen species production and/or decreased antioxidant enzyme activity, plays an important role in the pathogenesis of hypertension. The identification of molecular markers corresponding to the oxidative stress status of hypertension may assist in the antioxidant therapy of hypertension. In the present study, superoxide dismutase (SOD) and endothelial nitric oxide synthase (eNOS) were analyzed as markers of hypertension responding to oxidative stress. The plasma SOD activity and mononitrogen oxides (NO_x_) concentration were measured, and the SOD3 Ala58Thr and eNOS Glu298Asp polymorphisms were genotyped in hypertensive patients and normotensive controls. Further association experiments were replicated in an extended population, including 343 hypertensive patients and 290 controls. The results demonstrated that no statistically significant differences in the total SOD activity and NO_x_ concentration were identified between the hypertensive patients and controls. However, the plasma SOD activity levels in the SOD3 Ala/Ala homozygote carriers (80.51±27.68 U/ml) were significantly lower compared with the Thr allele carriers (92.18±16.37 U/ml; P=0.031). In addition, the plasma NO_x_ concentration in the eNOS Glu/Glu homozygote carriers (129.66±59.15 μmol/l) was significantly lower compared with the Asp allele carriers (169.84± 55.18 μmol/l; P=0.010). Notably, the altered SOD activity levels and NO_x_ concentration were in concordance in 56.3% of the 80 participants. Therefore, the concordance of decreased SOD activity and NO_x_ concentration, combined with genotypes of SOD3 Ala/Ala and/or eNOS Glu/Glu in hypertensive patients, may be useful in directing the antioxidant therapy of hypertension.

## Introduction

Hypertension is a major contributor to the development of cardiovascular, cerebrovascular and renal disease, and is influenced by genetic and environmental determinants. A large body of evidence indicates that endothelial dysfunction resulting from oxidative stress is a functional and reversible alteration of endothelial cells, which is characteristic of patients with hypertension (1–4).

Oxidative stress refers to an imbalanced redox status where pro-oxidants overwhelm the antioxidant capacity, resulting in the excessive generation of reactive oxygen species (ROS). Under physiological conditions, ROS function as important intracellular and intercellular secondary messengers to maintain the vascular function and integrity. However, under pathophysiological conditions, increased levels of ROS contribute to endothelial dysfunction and vascular remodeling. The superoxide anion (O_2_^−^) is the major ROS produced by endothelial and vascular smooth muscle cells, against which superoxide dismutase (SOD), as the first antioxidant defense system, protects to maintain the redox homeostasis. Three isoforms of SOD exist in mammalian cells, including cytoplasmic Cu/Zn SOD (SOD1), mitochondrial Mn SOD (SOD2) and extracellular SOD (SOD3). Among them, SOD3 is the major plasma SOD released from the tissue interstitium, which participates in scavenging O_2_^−^ (5). In addition, O_2_^−^ can react with nitric oxide (NO) to form peroxynitrite (ONOO^−^) that further oxidizes tetrahydrobiopterin (BH_4_), a cofactor of nitric oxide synthase (NOS), which leads to NOS uncoupling, producing O_2_^−^ and reducing the bioavailability of NO in the arteries (6,7). This dynamic alteration of ROS plays an important role in the pathogenesis of hypertension.

Based on the occurrence of oxidative stress in hypertension, the modification of antioxidant activity and NO bioavailability is considered an accessorial therapy for hypertension. Several antioxidant trials have been conducted with the aim of preventing or treating hypertension, including supplements of vitamin E, vitamin C or combinations. Although a few studies with a small sample size have demonstrated the beneficial effect of antioxidants in controlling hypertension, other larger trials have failed to confirm the effect of antioxidants on the development of hypertension or the control of blood pressure (8–12). These clinical trials have raised questions with regard to the oxidative stress status of hypertensive individuals as compared with normotensive controls. Furthermore, the correlation between plasma SOD activity and NO concentration has rarely been considered when interpreting the effect of antioxidant therapy in hypertension.

Thus, the aim of the present study was to evaluate SOD activity and NO concentration in hypertensive individuals and normotensive controls recruited from a rural area in Northeast China. The patients had not received any therapy. This relatively homogeneous population was useful for determining the redox status. In addition, associations between SOD activity and the Ala58Thr polymorphism (c.172G>A, rs2536512) in the SOD3 gene and mononitrogen oxides (NO_x_) concentration with the Glu298Asp polymorphism (c.894G>T, rs1799983) in the endothelial nitric oxide synthase (eNOS) gene were investigated.

## Methods

### Subjects

In total, 633 subjects of Han Chinese origin, including 343 unrelated hypertensive patients and 290 unrelated normotensive controls, were recruited from a relatively isolated rural area in Northeast China. Blood pressure (BP) was measured three times from the right arm of seated subjects after at least 5 min of rest. Hypertension was defined as an average systolic BP of ≥140 mmHg or an average diastolic BP of ≥90 mmHg. Patients were excluded from the study if they suffered from diabetes mellitus, renal disease or secondary hypertension, as determined by history inquiry and physical examination. All the individuals underwent routine laboratory tests that determined the levels of serum triglyceride (TG), total cholesterol (T-chol), high-density lipoprotein cholesterol (HDL-chol), low-density lipoprotein cholesterol (LDL-chol) and glucose. Informed consent was obtained from all the participants and ethical approval was provided by the local Ethics Committee (Shengjing Hospital of China Medical University, Shenyang, China).

### Measurement of plasma SOD activity and NO_x_ concentration

Plasma SOD activity and NO_x_ concentration were measured in 54 hypertensive patients and 26 normotensive controls, who were selected from the 633 subjects since they had not received antihypertensive therapy or other drugs. Measurements were performed using commercially available kits (No. A001-2/A013-1, Nanjing Jiancheng Bioengineering Institute, Nanjing, China), according to the manufacturer’s instructions. SOD activity was detected using chemical colorimetry, while the NO_x_ concentration was determined using nitrate reductase to convert nitrate (NO_3_^−^) to nitrite (NO_2_^−^), which was then detected by colorimetry.

### Genotyping

High-molecular weight genomic DNA was extracted from plasma buffy coats using a Flexigene kit (No. 51104, Qiagen GmbH, Hilden, Germany), according to the manufacturer’s instructions. Genotypes were determined using an Assays-on-Demand kit (No.C_2668728_10/C_3219460_20, Thermo Fisher Scientific, Foster City, CA, USA) in addition to *Taq*Man polymerase chain reaction (PCR) assays (No.4352042, Thermo Fisher Scientific). The probes and primers used in the *Taq*Man^®^ single nucleotide polymorphism (SNP) genotyping assays (C_2668728_10 for SOD3 Ala58Thr; C_3219460_20 for eNOS Glu298Asp) were purchased from Thermo Fisher Scientific. PCR was performed in a total volume of 25 μl per single well reaction in a 96-well plate containing 20 ng genomic DNA, 0.625 μl *Taq*Man^®^ SNP Genotyping Assay Mix (40X) and 12.5 μl *Taq*Man^®^ Universal PCR Master Mix (2X). The thermal cycling conditions were 95°C for 10 min, followed by 40 cycles of DNA denaturation at 92°C for 15 sec and annealing/extension at 60°C for 1 min. PCR was performed using the ABI 7500 Real-Time system (Thermo Fisher Scientific). DNA samples of known genotype and DNase-free water were used as positive controls and nontemplate controls in each assay run. Signals were processed and analyzed using the Sequence Detection System version 1.4 software package (Thermo Fisher Scientific). The samples genotyped Ala/Ala, Ala/Thr and Thr/Thr for SOD3 Ala58Thr, and Glu/Glu, Glu/Asp and Asp/Asp for eNOS Glu298Asp were selected randomly and confirmed by sequencing (3730 Genetic Analyzer; Thermo Fisher Scientific).

### Statistical analysis

Statistical analysis was performed with SPSS version 13.0 for Windows (SPSS, Inc., Chicago, IL, USA). Hardy-Weinberg equilibrium was assessed using the χ^2^ test. Clinical characteristics are presented as the mean ± standard deviation. Differences between two groups were assessed using the independent-sample t-test for quantitative variables and the χ^2^ test for categorical variables. Comparisons among three groups were performed by one-way analysis of variance. Adjusted odds ratios with 95% confidence intervals from the logistic regression analyses were used to estimate the relative risk for hypertension, while correlation analyses were used to determine the associations between quantitative variables. P<0.05 was considered to indicate a statistically significant difference.

## Results

### SOD activity in the hypertensive patients and controls

**Figure 1:**
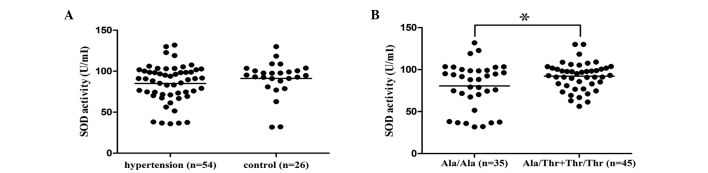
(A) Total plasma SOD activity in hypertensive patients and controls. (B) Association between the SOD3 Ala58Thr genotype and SOD activity. SOD activity levels in the Ala/Ala homozygote carriers were significantly lower than those in the Thr allele carriers (^*^P=0.031). SOD, superoxide dismutase.

Total SOD activity in the plasma embodies the antioxidant defense against O_2_^−^ and is predominantly attributable to SOD3, which exists exclusively in the extracellular space. Plasma SOD activity was assayed in 80 participants, including 54 hypertensive patients and 26 normotensive controls that had not undergone therapy. As shown in Fig. 1A, there was no statistically significant difference in the average levels of total SOD activity between the hypertensive patients and controls, however, the interindividual variability was more marked in the hypertensive patients than in the controls, indicating diverse susceptibilities of the antioxidant response to O_2_^−^ in hypertensive patients.

### Association between the SOD3 Ala58Thr polymorphism and SOD activity

The SOD3 Ala58Thr (c.172G>A) polymorphism at amino acid 40 in the amino terminal region of mature SOD3 is considered to be essential for tetramerization. In order to evaluate the association between the Ala58Thr polymorphism and plasma SOD activity, the SOD3 Ala58Thr polymorphism was genotyped using quantitative PCR in the same 80 participants whose plasma SOD activity levels were measured. As shown in Fig. 1B, plasma SOD activity in the SOD3 Ala/Ala homozygote carriers (80.51±27.68 U/ml) was significantly lower than in the Thr allele carriers (92.18±16.37 U/ml; P=0.031), indicating that the SOD3 Ala58Thr genotype was associated with decreased plasma SOD activity in a recessive model for the Ala allele.

### NO_x_ concentration in the hypertensive patients and controls

**Figure 2:**
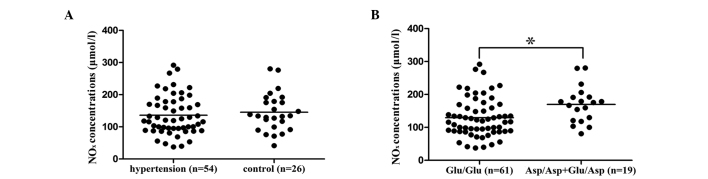
(A) Plasma NO_x_ concentration in the hypertensive patients and controls. (B) Association between the eNOS Glu298Asp genotype and NO_x_ concentration. NO_x_ concentrations in the Glu/Glu homozygote carriers were significantly lower than in the Asp allele carriers (^*^P=0.010). NO_x_, mononitrogen oxides; eNOS, endothelial nitric oxide synthase.

NO released from the cells was rapidly oxidized to NO_2_^−^ and NO_3_^−^, termed NO_x_. These molecules are relatively stable in blood, thus, the plasma NO_x_ concentration may be an indicator of the endogenous formation of NO. The median plasma NO_x_ concentration in the hypertensive patients (136.2 μmol/l; interquartile range, 37.6–291.7 μmol/l) was not significantly different from the controls (145.4 μmol/l; interquartile range, 41.4–280.6 μmol/l). However, there was a decreased tendency in NO_x_ concentration in the hypertensive patients, indicating more frequent occurrence of eNOS uncoupling (Fig. 2A).

### Association between the eNOS Glu298Asp polymorphism and NO_x_ concentration

To identify whether the eNOS Glu298Asp (c.894G>T) polymorphism was associated with the plasma NO_x_ concentration, the Glu298Asp polymorphism was genotyped in 80 participants whose plasma NO_x_ concentration had been detected. As shown in Fig. 2B, the plasma NO_x_ concentration in the eNOS Glu/Glu homozygote carriers (129.66±59.15 μmol/l) was significantly lower compared with the Asp allele carriers (169.84±55.18 μmol/l; P=0.010), indicating that the eNOS Glu298Asp genotype was associated with decreased plasma NO_x_ concentrations in a recessive model for the Glu allele.

### Concordance of SOD and NO_x_ in hypertensive patients and controls

Concordance of SOD and NO_x_ refers to the uniform alteration of SOD activity and NO_x_ concentration in each individual. As shown in Table I, 56.3% of the 80 participants were in concordance, among them, 23 subjects had above average values of SOD and NO_x_, while 22 subjects had below average values. The individuals with below average values were predominantly hypertensive patients (17/22), of which the SOD3 Ala/Ala and/or eNOS Glu/Glu genotype accounted for 88.2%.

### Association study in a case-control population

Considering the associations between SOD3 Ala58Thr and plasma SOD activity and between eNOS Glu298Asp and NO_x_ concentration, the association between the polymorphisms and hypertension was investigated in a case-control population, including 343 hypertensive patients and 290 normotensive controls. As shown in Table II, serum TG, T-chol, LDL-chol, potassium and sodium levels were higher in the hypertensive patients compared with the controls; whereas, HDL-chol was lower in the hypertensive patients compared with the controls. Correlation analysis revealed positive associations between serum TG, T-chol, LDL-chol and glucose with BP; whereas, serum HDL-chol and potassium were shown to be negatively associated with BP.

Genotype distributions of SOD3 Ala58Thr and eNOS Glu298Asp were in Hardy-Weinberg equilibrium. As shown in Table III, neither genotype nor allele frequency was shown to be significantly different between the hypertensive patients and normotensive controls.

## Discussion

Oxidative stress is characterized by increased ROS production and/or decreased antioxidant enzyme activity. SOD is the predominant antioxidant enzyme that protects the cell from oxidative damage by converting O_2_^−^ to hydrogen peroxide. Plasma SOD activity levels have been found to be altered in a number of diseases, including ovarian cancer (13), idiopathic respiratory distress syndrome and colorectal cancer (14,15), and are considered to be a good marker for detecting and monitoring these diseases. However, data on the change of SOD activity in experimental and human hypertension studies are inconsistent. In spontaneously hypertensive rats, SOD3 activity levels was observed to be significantly decreased (16), and overexpression of SOD3 reduced systemic vascular resistance and arterial pressure (17). In humans, plasma SOD activity was reported to be significantly reduced when compared with normotensive controls (18,19). However, certain studies have reported no change or higher plasma SOD activity with hypertension (20,21). In the present study, the plasma SOD activity levels were assessed in hypertensive patients for further use as a diagnostic or healing marker. Plasma SOD activity was measured in 80 subjects that had not received therapy, including 54 hypertensive patients and 26 normotensive controls, from a relatively homogeneous population. No statistically significant difference was observed in the average plasma SOD activity between the hypertensive patients and controls, however, the interindividual variability of SOD activity was more marked in the hypertension patients than in the controls. This variation may have been caused by the SOD3 polymorphisms that have been widely studied (22,23). One functional variant, a Gly213 substitution for Arg213 (Arg213Gly) at the heparin-binding domain of SOD3, results in a 10-fold increase in plasma SOD3 activity levels and a decrease in tissue SOD3 activity levels (24). High plasma activity can be explained by an accelerated release from the tissue interstitium. However, the Gly213 carriers account for 4% of Swedish, 3% of Australian and 6% of Japanese populations that have been studied (25–27), and SOD activity variation cannot be ascribed to the relatively rare variant. The SOD3 Ala58Thr polymorphism is located at amino acid 40 in the amino terminal region of mature SOD3 and is considered to be essential for tetramerization. However, little is known with regard to the effect of Ala58Thr on SOD activity. Thus, the SOD3 Ala58Thr polymorphism was further genotyped to identify whether there was an association between the genotype and plasma SOD activity. The Ala/Ala homozygote carriers were found to have decreased SOD activity levels when compared with the Thr allele carriers. However, no difference between this polymorphism and SOD activity was found in a Japanese population (28). This difference may be caused by the different study populations.

SOD3 is potentially involved in the mechanism responsible for the impairment of plasma NO bioavailability. In endothelial cells, NO is predominantly produced from L-arginine by eNOS. Under normal conditions, the active form of eNOS is a homodimer and exhibits an antihypertensive effect via the function of NO, which inhibits platelet adhesion to the endothelium and relaxes the vascular smooth muscle. However, in the absence of the substrate L-arginine or BH_4_, eNOS exists in an inactive monomer form and produces O_2_^−^ rather than NO. Furthermore, O_2_^−^ can react with NO to form a potent oxidizing agent, peroxynitrite (ONOO^−^), leading to the reduction of NO bioavailability and endothelial dysfunction. Therefore, eNOS may exhibit an anti- or a prohypertensive effect (29). In the present study, no statistically significant difference in NO_x_ concentration was observed between the hypertensive patients and controls, however, there was a decreased tendency in NO_x_ concentration in the hypertensive patients. The results were in accordance with the study by Node *et al* (30) that reported that the plasma NO concentration was reduced in patients with hypertension. However, Sandrim *et al* (31) found that NO_x_ concentrations were increased in hypertensive patients, indicating that the increased NO level may play a compensatory role. With regard to eNOS polymorphisms, three have been widely studied: 786T/C in the promoter region, a 27-bp variable number of tandem repeats in intron 4 (intron4b/a) and Glu298Asp (c.894G>T) in exon 7. The Glu298Asp polymorphism is located between the critical residue of the heme domain and the binding sites for L-arginine and BH_4_ (32). Therefore, the substitution of Glu by Asp may result in an alteration of eNOS activity and plasma NO production. Association studies between eNOS Glu298Asp and plasma NO_x_ concentration have produced inconsistent results (33–37). In the present study, the Glu/Glu homozygote carriers had significantly lower NO_x_ concentrations than the Asp allele carriers, which was in accordance with the study by Yoon *et al* (36). However, an association between the polymorphism and eNOS expression or eNOS enzyme activity was not observed in cultured umbilical vein endothelial cells in their subsequent study (37). Metzger *et al* demonstrated no variation in NO_x_ concentration across the genotypes (38). Therefore, the effect that the eNOS Glu298Asp polymorphism has on plasma NO_x_ concentration in the population of the present study may require further identification using larger test samples.

SOD regulates the bioavailability of NO via the reduction of O_2_^−^, which reacts with NO. Due to the interaction of SOD and NO in the plasma, their concordance was calculated to evaluate the individual redox status. Of the 80 tested subjects, 56.3% exhibited good concordance and the sum of the individuals with above average values of SOD activity and NO_x_ concentration (n=23) was close to those with below average values (n=22). Individuals with SOD and NO_x_ below average values were primarily hypertensive patients carrying the SOD3 Ala/Ala and/or eNOS Glu/Glu genotypes, which coincided with the association of phenotype with SOD3 and eNOS genotypes. Therefore, the concordance of plasma SOD activity and NO_x_ concentration combined with their genotypes was hypothesized to be valuable to antioxidant trials of hypertension. In addition, an extended case-control population was genotyped, including 343 hypertensive patients and 290 unrelated controls from the same region in Northeast China. However, an association between SOD3 Ala58Thr and hypertension was not identified. Thus far, studies on the association between human SOD3 Ala58Thr and hypertension have produced inconsistent results, with significant positive associations in specific studies (39–41), but not in others (42). This inconsistency may derive from population stratification of ethnicity. In the present case-control population, Ala was the major allele of SOD3 Ala58Thr (68.5%), which is the same as Spanish (63%) (41) and Japanese (71.0%) (42) populations, but Thr is the major allele in a Romanian (67.0%) population (40). With regard to the eNOS Glu298Asp polymorphism, the association with hypertension is also controversial. Miyamoto *et al* reported that the Asp allele was associated with hypertension in a Japanese population (43) and Lacolley *et al* found that the Glu allele was associated with hypertension in Caucasians (44). However, other studies have not replicated the association between eNOS Glu298Asp and hypertension in Japanese, Caucasian and African American populations (45–47). In the present study, an association between eNOS Glu298Asp and hypertension was not observed in the case-control population, confirming the results of a previous study that reported that the polymorphism was unlikely to be a major genetic susceptibility factor for hypertension in the north Han Chinese population (48).

In conclusion, the present study demonstrated an association between the SOD3 Ala58Thr polymorphism and plasma SOD activity, as well as an association between the eNOS Glu298Asp polymorphism and plasma NO_x_ concentrations in a Northeastern Chinese population. Ala/Ala homozygote carriers at the SOD3 Ala58Thr locus had decreased plasma SOD activity levels, while the Glu/Glu homozygote carriers at the eNOS Glu298Asp locus had decreased plasma NO_x_ concentrations. The concordance between decreased SOD activity and NO_x_ concentration, combined with the genotypes of SOD3 Ala/Ala and/or eNOS Glu/Glu in hypertensive patients, may be useful in directing the antioxidant therapy of hypertension.

## Figures and Tables

**Table I:** Concordance of SOD activity and NO_x_ concentration in HTN patients and controls.

	Concordance		
	
SOD or NO_x_	Total, n (%)	HTN, n (%)	Control, n (%)
Above average	23 (28.8)	13 (16.3)	10 (12.5)
Below average	22 (27.5)	17 (21.2)	5 (6.3)

HTN, hypertensive; SOD, superoxide dismutase; NO_x_, mononitrogen oxides.

**Table II:** Baseline characteristics of the case-control population.

Characteristics	HTN (n=343)	Control (n=290)	P-value
Male/female, n	146/197	116/174	0.515
Age, years	48.23±9.15	47.46±8.43	0.277
SBP, mmHg	166.78±20.18	113.29±9.19	<0.001
DBP, mmHg	104.34±12.00	74.43±6.26	<0.001
TG, mmol/l	1.58±1.17	1.14±1.05	<0.001
T-chol, mmol/l	5.07±0.97	4.72±1.01	<0.001
LDL-chol, mmol/l	3.05±0.76	2.73±0.79	<0.001
HDL-chol, mmol/l	1.52±0.37	1.67±0.50	<0.001
Glucose, mmol/l	4.83±1.50	4.63±1.40	0.097
Potassium, mmol/l	4.15±0.40	4.23±0.38	0.011
Sodium, mmol/l	144.32±3.75	143.61±4.28	0.026

Data are expressed as the mean ± SD. HTN, hypertensive; SBP, systolic blood pressure; DBP, diastolic blood pressure; TG, triglyceride; T-chol, total cholesterol; LDL-chol, low-density lipoprotein cholesterol; HDL-chol, high-density lipoprotein cholesterol.

**Table III:** Genotype and allele distributions of SOD3 Ala58Thr and eNOS Glu298Asp in the case-control population.

Parameter	HTN, n (%)	Control, n (%)	OR (95% CI)	P-value
*SOD3* Ala58Thr				
Genotype				
Ala/Ala	158 (46.1)	132 (45.5)		
Ala/Thr	154 (44.9)	130 (44.8)		0.963
Thr/Thr	31 (9.0)	28 (9.7)		
OR^a^			0.930 (0.544–1.590)	0.790
OR^b^			0.978 (0.715–1.339)	0.891
Allele				
Ala	470 (68.5)	394 (67.9)		
Thr	216 (31.5)	186 (32.1)		
OR			1.027 (0.810–1.302)	0.825
*eNOS* Glu298Asp				
Genotype				
Glu/Glu	257 (74.9)	224 (77.2)		
Glu/Asp	81 (23.6)	64 (22.1)		0.573
Asp/Asp	5 (1.5)	2 (0.7)		
OR^a^			2.130 (0.410–11.062)	0.357
OR^b^			1.136 (0.787–1.640)	0.497
Allele				
Glu	595 (86.7)	512 (88.3)		
Asp	91 (13.3)	68 (11.7)		
OR			0.868 (0.621–1.215)	0.410

a In a dominant model for *SOD3* Ala or *eNOS* Glu;

b In a recessive model for *SOD3* Ala or *eNOS* Glu.

HTN, hypertensive patients; OR, odds ratio; 95% CI, 95% confidence interval; SOD, superoxide dismutase; eNOS, endothelial nitric oxide synthase.
